# Impacts of sub-micrometer sediment particles on early-stage growth and survival of the kelp *Ecklonia bicyclis*

**DOI:** 10.1038/s41598-020-75796-x

**Published:** 2020-11-26

**Authors:** Akira Matsumoto, Minami Sato, Hisayuki Arakawa

**Affiliations:** 1 Tokyo University of Marine Science and Technology, 4-5-7 Konan, Minato-ku, Tokyo, 108-8477 Japan; 2 Fukushima Prefectural Research Institute of Fisheries Resources, 1-1-14 Koyo, Soma, Fukushima 976-0005 Japan

**Keywords:** Plant sciences, Ecology, Environmental sciences

## Abstract

Marine forests have declined in many urbanized regions in recent years. One cause is the inflow of fine particles into coastal rocky shores. We examined the influence of sub-micrometre (sub-micro) particles on the early growth stages of the large brown macrophyte *Ecklonia bicyclis*. The percentage of substrate attachment of zoospores decreased with an increase in sub-micro sediments. As the size of the particles decreased, the negative effect became greater. There was an increase in poor levels of gametophyte survival and growth as more and smaller sediment was deposited. We consider that the causes of these phenomena owing to increasing amounts of sediment is a decrease in availability of substrate for zoospore attachment and that of area for substance exchange on the gametophytes. We also evaluated the effects in sea areas, based on the amount and size distribution of seabed sediment in the algal communities deforested by particles, and found that the inhibition of zoospore attachment and gametophyte growth by sub-micro particles was remarkably large. The sub-micro sediment on the substrate has seriously negative effects on the early stages of macrophytes. Inflow of very fine particles to natural marine forests may result in severe degradation of rocky reef ecosystems.

## Introduction

Communities composed of large brown algae are major primary producers and maintain coastal ecosystems^1,2^. However, deforestation of these marine communities has been reported all over the world^3,4^. This deforestation is considered to be the result of high seawater temperatures^5,6^, inappropriate nutrient levels^5,7,8^, subsidence^9^, feeding pressure from herbivores^10–15^, competition with other species of algae^2,16^ and the increase in particles in the sea area^17^. These phenomena are the cause of deforestation by acting alone or in combination.

The increase in particles in the sea area causes an increase in the concentration of suspended particles in seawater and/or sediment particles on the seabed. The amount and distribution of sediment particles on the seabed change because of natural phenomena such as runoff from rivers and coastal waves, and anthropogenic activities such as dredging and construction. When the sediment particles increase in the kelp forest, the canopy is replaced by turf-forming communities^3,17–19^. The increased sediment particles on rocky reefs negatively impact the early growth stage of kelp communities^20–24^, and the influence of smaller particles is remarkably large^25,26^. Watanabe et al.^26^ examined the influence of different sizes of particles (15–599 µm) on the early growth stage of the large brown alga *Ecklonia bicyclis*. The influence of smaller particles on zoospore substrate attachment and gametophyte survival was found to be greater than that of larger particles.

Mineral and/or organic particles of sub-micrometer (sub-micro) size (nearly 1 µm) are distributed throughout the oceans^27,28^. Many particles that are sub-micro in size are actively produced by various industrial processes^29,30^, and there is concern that some of these may leak into the environment. Although the details of their quantity and distribution in the sea are unknown, laboratory experiments have reported that fine particles have negative effects on marine organisms^31–33^. These studies have mainly examined the toxicity of particles to organisms, resulting from their chemical composition, and there are no reports examining the effects on macrophytes resulting from their physical properties.

This study used laboratory experiments to estimate the impact of fine sedimentary particles in the field on the early stage of macrophyte growth, and the influences of sub-micro size sedimentary particles on zoospore attachment to the substrate and on gametophyte growth and survival. We also used these results to evaluate the effects of sub-micro particles on the initial depletion of *E. bicyclis* in sea areas in Mio, Wakayama Prefecture, Japan, where kelp communities were deforested by particles.

## Results

### Influence on zoospore attachment

A slide glass with various sub-micro particles was deposited in a container (outer diameter 61.8 mm, height 125.2 mm) filled with seawater. Zoospores were poured from the surface of the water, and the number of zoospores that had attached to the slide glass was counted. The effect of the particles on attachment was investigated. Here, particles A, B and C were used (silicon carbide–SiC–particles with different size distributions) as the sediment particles. Particles A and B had one peak in the size distribution, and average particle sizes of 1.1 µm and 3.9 µm, respectively. Particle C had two peaks at 0.090 µm and 4.6 µm, and the average particle size was 1.5 µm (Supplementary Fig. S1 online).

**Figure 1:**
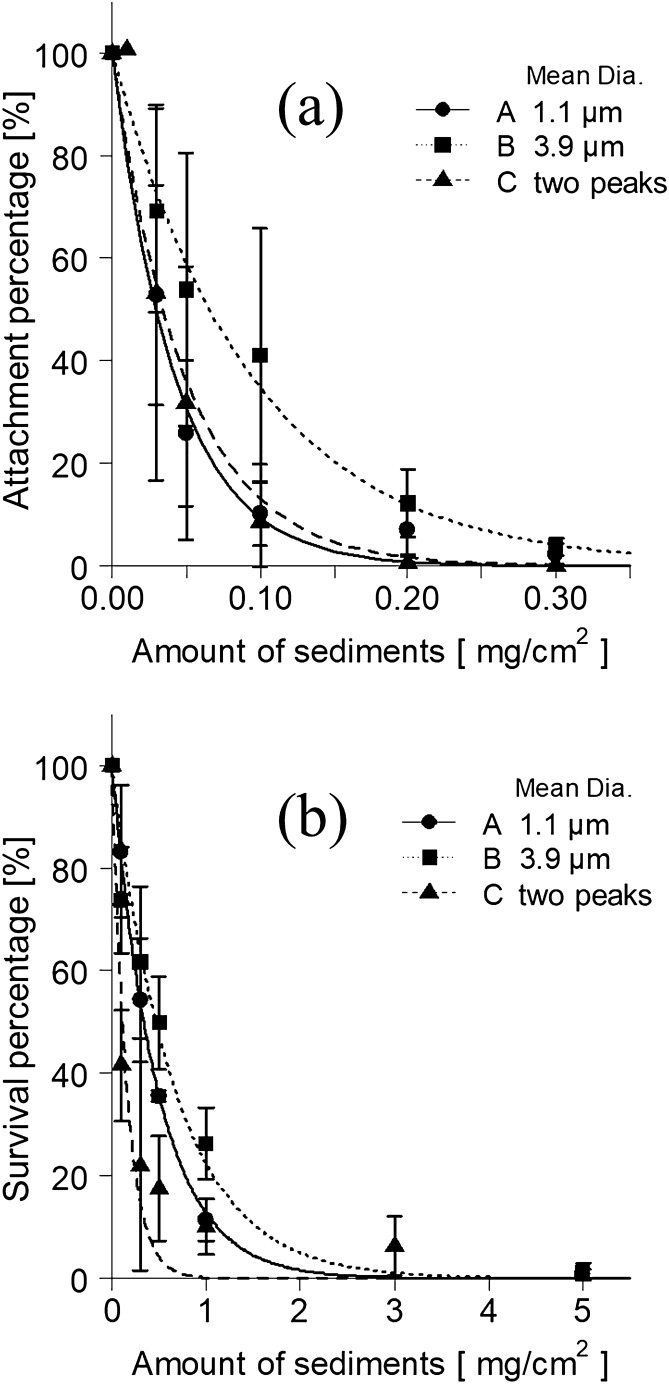
Negative influences of sediment on zoospore attachment and gametophyte survival; (**a**,**b**) zoospore attachment percentage and gametophyte survival percentage, respectively.

When about 5 × 10^4^ of *E. bicyclis* zoospores were placed in the container, after 12 h an average attachment of 13.5 ind./mm^2^ was observed on the slide glass without sediment particles. The relationship between the attachment percentage (%) of zoospores and amount of sediment particles of SiC is shown in Fig. 1a. The attachment percentage, expressed as the number of attached zoospores without sediments, was 100%.

In the case of particle A (mean diameter 1.1 µm), which had one peak in the size distribution, the zoospore attachment percentage (mean ± SD) at 0.05 mg/cm^2^ and 0.1 mg/cm^2^ of sediments were 25.9 ± 14.2% and 10.2 ± 6.17%, respectively (Fig. 1a, Supplementary Table S1 online). In the case of particle B (mean diameter 3.9 µm), the attachment percentage was 53.9 ± 24.8% at 0.05 mg/cm^2^ and 41.1 ± 23.1% at 0.1 mg/cm^2^. In the case of particle A, few attachments were found at sediment levels of 0.3 mg/cm^2^.

The attachment percentage decreased exponentially as the amount of sediment on the substrate increased at any particle size. A significant negative correlation (Spearman’s rank correlation, *p* < 0.05, *rs* = − 1 and − 1 for particles A and B, respectively) was observed between the amount of sediment and the attachment percentage for each particle size. For each particle, the approximate expression by the nonlinear least squares method and the pseudo-determination coefficient were Eqs. (1) and (2):

1 $$ {\text{Particle A }}\left( {{\text{mean diameter 1.1}}\,\upmu {\text{m}}} \right)\;A_{r} { = 100}\;{\exp}\left( - 23.7\,Q \right),\quad \left( {r^{2} = 0.{988}} \right) $$

2 $$ {\text{Particle B }}\left( {{\text{mean diameter 3}}.{9}\,\upmu {\text{m}}} \right)\;A_{r} { = 100}\;{\exp}\left( { - {10}{\text{.6}}\,Q} \right),\quad \left( {r^{2} = 0.{988}} \right) $$

where *A*_*r*_ is the zoospore attachment percentage (%) and *Q* is the amount of sediment (mg/cm^2^).

In the case of particle C, with two peaks in particle size distribution, the attachment percentage was 31.8 ± 25.8% at 0.05 mg/cm^2^ and 8.55 ± 10.9% at 0.1 mg/cm^2^, and it became less than 1% at 0.2 mg/cm^2^. The attachment percentage for particle C decreased exponentially as the amount of sediment on the substrate increased. The change in particle C was similar to that of particle A. We found a significant negative correlation (Spearman’s rank correlation, *p* < 0.05, *rs* = − 0.964) between the amount of sediment and the attachment percentage. The approximate expression of the nonlinear least squares method and pseudo-determination coefficient for the relationship between the zoospore attachment percentage and the amount of sediment in particle C is shown in Eq. (3):

3 $$ {\text{Particle C }}\left( {\text{two peaks}} \right)\;\;A_{r}  = 100{\text{  exp}}\left( { - {20}{\text{.2 }}Q} \right),\quad \left( {r^{2} = 0.962} \right) $$

### Influence on survival and growth of gametophytes

The slide glass on which the zoospores were attached was set in a Petri dish (diameter: 150 mm, height: 90 mm) filled with seawater. Various amounts of fine particles were deposited on the slide glass, and the influence of the sediment particles on the growth and survival of gametophytes was examined. Here, particles A, B and C, which are the same as those used in the attachment experiment, were used as the sediment particles.

The relationships between the survival percentage of the gametophytes and the amount of sediment are shown in Fig. 1b. The survival of gametophytes placed in Petri dishes with sediments is expressed as a percentage of the gametophytes surviving 12 days in Petri dishes without sediments.

When particle A (mean diameter 1.1 µm) with one peak in particle size distribution was deposited, the survival percentages (mean ± SD) of the gametophyte were 83.2 ± 12.9% at 0.1 mg/cm^2^ and 11.4 ± 4.04% at 1.0 mg/cm^2^ sediments. The survival percentage decreased remarkably as sediment amounts increased. The survival percentages for particle B (mean diameter 3.9 µm) were 73.8 ± 10.3% at 0.1 mg/cm^2^, and 26.4 ± 7.04% at 1.0 mg/cm^2^. The survival percentage decreased exponentially as the amount of sediment on the gametophyte increased. In the cases of particles A and B, there was a significant negative correlation (Spearman’s rank correlation, *p* < 0.05, *rs* = − 1 and − 1 for particles A and B, respectively) between the amount of sediment and the survival percentage. The relational expression approximated by the nonlinear least squares method and the pseudo-determination coefficient is shown in Eqs. (4) and (5):

4 $$ {\text{Particle}}\;{\text{A}}\;\left( {{\text{mean}}\;{\text{diameter}}\, 1.1\,\upmu {\text{m}}} \right) \;\;S_{r} = 100\;\exp \left( { - 2.06\,Q} \right),\quad \left( {r^{2} = 0.999} \right) $$

5 $$ {\text{Particle}}\;{\text{B}}\;\left( {{\text{mean}}\;{\text{diameter}}\;3.9\,\upmu {\text{m}}} \right) \;\;S_{r} = 100 \exp \left( { - 1.49 Q} \right),\quad \left( {r^{2} = 0.970} \right) $$

where *S*_*r*_ is the survival percentage of gametophytes (%) and *Q* is the amount of sediment (mg/cm^2^).

In contrast, the survival percentage of gametophytes in the case of particle C sediment (mean diameter 1.5 µm) with two peaks in the particle size distribution was 41.6 ± 10.8% at 0.1 mg/cm^2^ and 10.2 ± 5.38% at 1.0 mg/cm^2^. The negative effect of particle C on survival was greater than the effect of the same amount of particles A and B. In particle C, a significant negative correlation (Spearman’s rank correlation, *p* < 0.05, *rs* = − 1) was observed between the amount of sediment and the survival percentage. The relational expression approximated between the survival percentage of the gametophyte and the amount of sediment by the nonlinear least squares method and the pseudo-determination coefficient is shown in Eq. (6):

6 $$ {\text{Particle C }}\left( {\text{two peaks}} \right)\;\;S_{r} = 100 \exp \left( { - 6.31 Q} \right),\quad \left( {r^{2} = 0.919} \right) $$

where *S*_*r*_ is the survival percentage of gametophytes (%) and *Q* is the amount of sediment (mg/cm^2^).

The culture of *E. bicyclis* gametophytes was performed with sediment of each particle size, and the total length of the gametophytes was measured on the 6th and 12th days. The total length of gametophytes without sediments was 37.7 ± 10.5 µm on day 6, and was 94.1 ± 26.0 µm for female and 137 ± 27.4 µm for male on day 12. The total length of the gametophytes on the 12th day, when particles of all sizes had been deposited, is shown in Table 1.

**Table 1:** Total length of gametophyte at 12 days under each sediment particle treatment.

Mean particle diameter (µm)		Amount of sediments (mg cm^−2^)	Male			Female		
			N	Body length (%)	SD	N	Body length (%)	SD
A	1.1	0	124	100	–	118	100	–
		0.1	124	91.6	17.9	125	91.7	15.6
		0.3	117	87.8	18	118	86.9	18.3
		0.5	119	82.4	21.3	96	75	16.6
		1.0	65	69.5	18.3	40	66.7	19.8
B	3.9	0	125	100	–	125	100	–
		0.1	125	88.9	15.8	126	82.5	15.7
		0.3	125	89.6	15.6	125	81.7	18.2
		0.5	125	80.0	17.0	125	75.8	16.7
		1.0	125	80.7	18.3	125	71.7	24.3
		5.0	37	78.5	15.6	37	68.3	20.2
C	Two peaks	0	126	100	–	126	100	–
		0.1	126	91.1	22.4	126	92.3	18.8
		0.3	97	89.6	21.2	90	83.3	18.2
		0.5	126	83.0	21.6	109	80.8	15.1
		1.0	116	77.0	20.7	90	74.4	15.3
		3.0	19	63.0	21.7	5	71.8	14.6

If the total length on the 12th day with no sediments was 100%, the relative total length for particles A (mean diameter 1.1 µm) in 0.1 and 1.0 mg/cm^2^ sediment amounts were 91.7 and 66.7% for female gametophytes, and 91.6 and 69.5% for male gametophytes, respectively. When the sediment amount was 5 mg/cm^2^, the gametophytes did not grow to a size that enabled discrimination between male and female. With respect to particle B (mean diameter 3.9 µm), the total length at sediment volumes of 0.1 and 1.0 mg/cm^2^ were 82.5 and 71.7% for female gametophytes, and 88.9 and 80.7% for male gametophytes, respectively. Gametophyte growth was worse as the amount of sediment increased, and the negative effect was greater when the particle size of the sediment was small. In particle C with two peaks, the total length at sediment volumes of 0.1 and 1.0 mg/cm^2^ were 92.3 and 74.4% for female gametophytes, and 91.1 and 77.0% for male gametophytes, respectively. When the sediment volume was 3.0 mg/cm^2^, the total lengths of female and male gametophytes were 71.8% and 63.0%, respectively.

Covariance analysis (ANCOVA) was performed on the total length as a response variable. Among the explanatory variables, particle size (mean diameter) and sediment volume were treated as continuous variables, and the sex of the gametophyte was treated as a categorical variable. It was shown that the growth of gametophytes was inhibited for each volume of sediment and at each particle size (*p* < 0.001; Table 2). The total length of female gametophytes was significantly smaller than that of males (*p* < 0.001). There was an interaction between particle size and sex (*p* < 0.001) (Fig. S2a), and the negative effect was found to be strong in female gametophytes. On the other hand, there was no interaction between sediment amount and sex (*p* = 0.215) (Fig. S2b).

**Table 2:** ANCOVA results (Type 3 sum of squares) of the effects of sediment quantity and particle size on male and female gametophyte growth.

Source	df	MS	F-value	P value
**Gametophyte total length**				
Particle size	1	331,971	558.552	< 0.001
Amount of sediments	1	56,570	95.182	< 0.001
Sex	1	701,326	1180.003	< 0.001
Particle size × sex	1	106,638	179.422	< 0.001
Amount of sediments × sex	1	915	1.54	0.215
Residuals	3497	2078,417		

### Effects of gaps on substrate on zoospore attachment and gametophyte survival

We examined the relationship between the space on the substrate not covered by particles (the ‘percentage gap’) and zoospore attachment percentage, and the relationship between the percentage gap and gametophyte survival percentage. The percentage gap (%) is the ratio of the gap area to the substrate area (the ratio of voids between particles).

**Figure 2:**
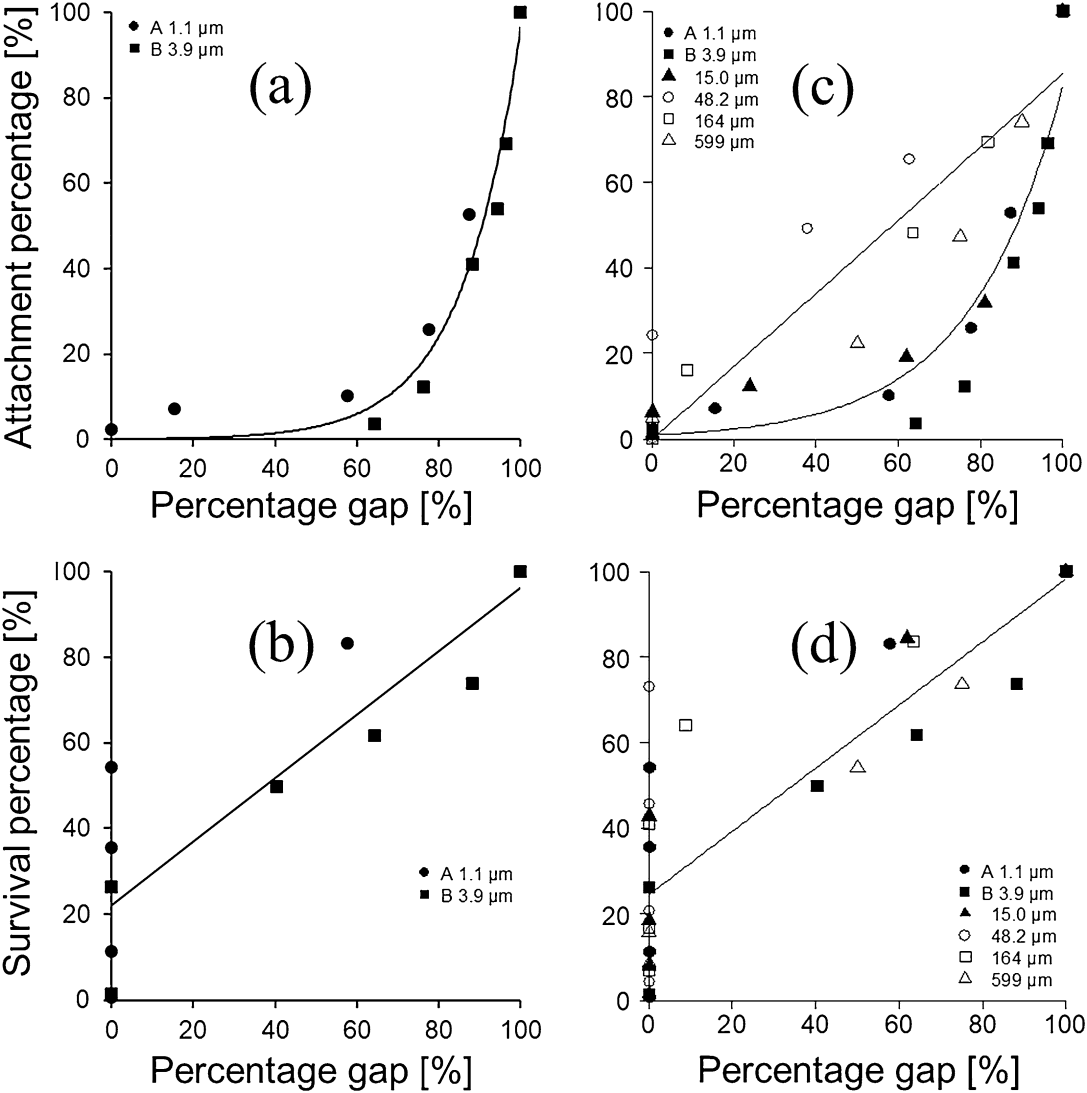
Relationship between percentage gap on substrate and attachment percentage of zoospore (**a**) or survival percentage of gametophyte (**b**). Data of particle size 15.0–599 µm are shown from the relational expression of Watanabe et al.^26^ in (**c**,**d**).

In the case of 1.1 and 3.9 µm particles, the attachment percentage decreased exponentially with decreasing percentage gap (Fig. 2a). In both particle size ranges, when the percentage gap was 0%, the zoospore attachment percentage was almost 0%. However, in the 48.2–599 µm particle range^26^, the attachment percentage decreased linearly as the percentage gap decreased (Fig. 2c). This result suggested that the attachment percentage of zoospores was governed by the percentage gap on the substrate.

The survival percentage of gametophytes deposited on 1.1 and 3.9 µm particles tended to decrease linearly as the percentage gap decreased (Fig. 2b). In the case of the 48.2–599 µm particle range, the survival percentage indicated the same trend in decrease (Fig. 2d). This change was different from the case of the attachment percentage, and showed the same change regardless of the particle diameter. However, even when the percentage gap was 0%, the survival percentage indicated a range of 0% to ca 70%, and the survival percentage decreased as the sediment thickness increased.

### Sediment amount and size distribution in kelp communities deforested by sediment particles

The large kelp communities off Mio, Wakayama Prefecture, Japan, have been deforested owing to turbidity caused by Hidakagawa River inflow in the 1990s^34^. We measured the amount and size distribution of seabed sediment particles off Mio, and also in nearby off Noshima where the kelp communities are maintained (Supplementary Fig. S3 online).

**Figure 3:**
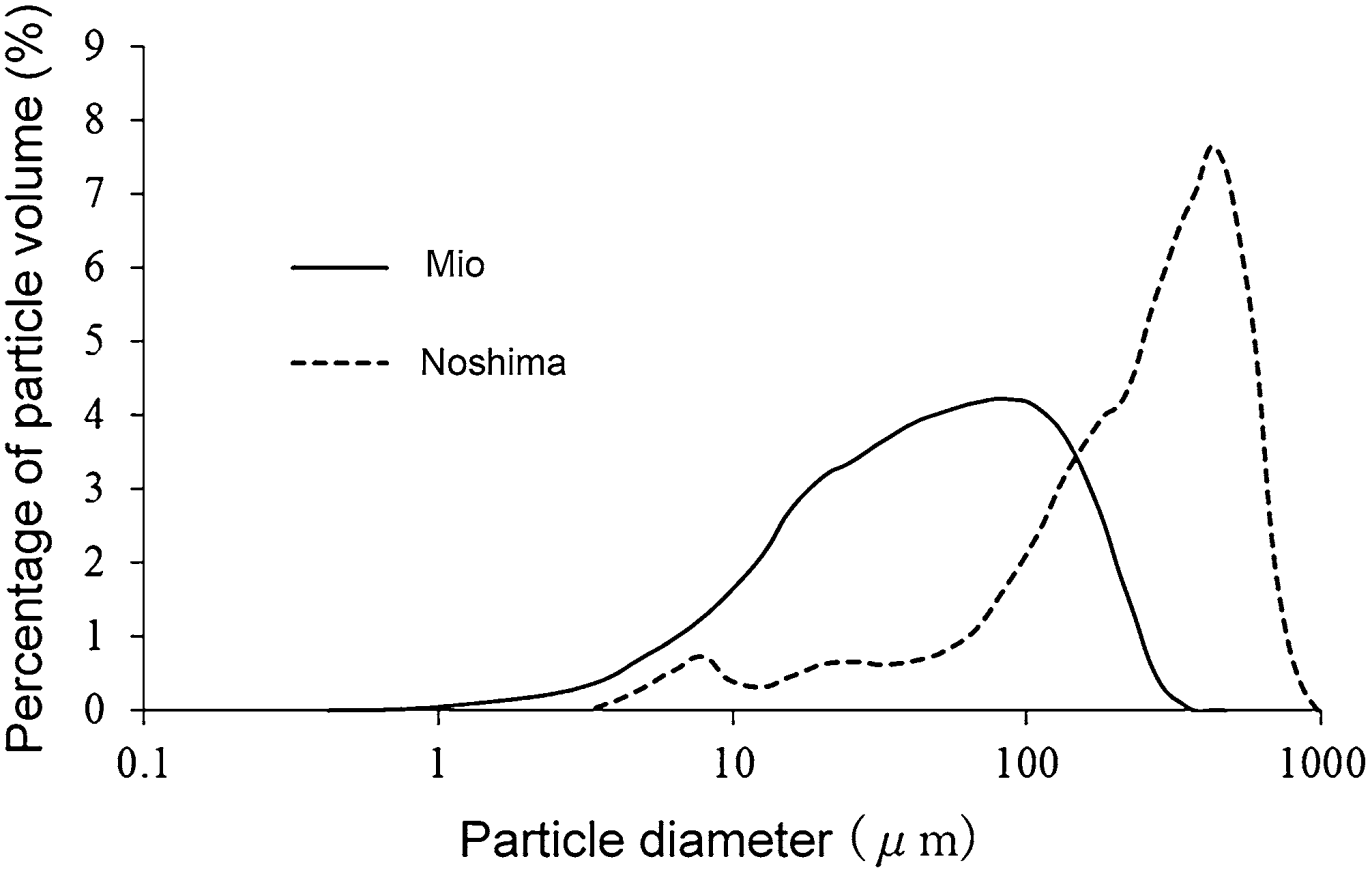
Particle size distribution of seabed sediments in deforested sea area.

The amount of sediment particles in Mio and Noshima was 2.03 ± 0.643 mg/cm^2^ and 2.07 ± 1.95 mg/cm^2^, respectively, and no significant difference was observed (Student-t test; *P* > 0.05). Figure 3 shows the particle size distribution of sediment particles in both seas. The mode and the average particle size of distribution in Mio were 79.3 µm and 39.1 µm, respectively. In contrast, the mode and average particle size in Noshima were 420 µm and 185 µm, respectively. Assuming that the sub-micro size is less than 10 µm, particles of that size were found at a level of 10.1% in Mio compared to 3.7% in Noshima.

## Discussion

In this study we investigated the effects of sub-micro sedimentary particles (mean diameter 1.1 µm and 3.9 µm) on zoospore attachment to the substrate and on the growth and survival of gametophytes. Watanabe et al.^26^ investigated particles of 15–599 µm diameter.

According to Watanabe et al. the attachment percentage was 31.8% when the amount of sediment measuring 15 µm mean diameter was 1 mg/cm^2^. In contrast we found that the attachment percentage of particle A (1.1 µm diameter) was 10.2% when the sediment volume was 0.1 mg/cm^2^. The negative effect of sediment having a particle size of ca. 1 µm was remarkably greater than that of sediment with a particle size of 15 µm.

The survival percentage was 64.2% when the amount of sediment measuring 15 µm mean diameter was 1 mg/cm^2^. With particle A, the survival percentage was 11.4% when the sediment volume was 1 mg/cm^2^.

The negative effect on substrate attachment was greater than the effect on gametophyte survival. We especially found that the negative influence of sub-micro seabed sediments on the zoospore attachment of large brown macrophytes is notably large.

A review by Airoldi^17^ reported that sedimentary particles on the seabed affect the ecology and distribution of algae. However, few reports quantitatively analyse the effects of sediment particles^18,22,26^. Devinny and Volse^35^ reported that *Microcystis pyrifera* spores do not selectively attach to hard, large substrates, but are also attached to sediments, so they are swept away by water movement. Park and Hwang^36^ noted that sediments with small particles reduce the areas on a substrate to which migrating cells can attach, inhibiting their attachment there.

In the 48.2–599 µm particle range^26^, the attachment percentage decreased linearly as the percentage gap decreased (Fig. 2c). However, in the case of 1.1–15.0 µm particles, the attachment percentage decreased exponentially with decreasing percentage gap.

The survival percentage of gametophytes tended to decrease linearly as the percentage gap decreased (Fig. 2d). This change was different from that of the attachment percentage, and showed the same change regardless of the particle diameter. However, even when the percentage gap was 0%, the survival percentage indicated a range of 0% to ca 70%, and the survival percentage decreased as the sediment thickness increased.

There were two trends in the decrease in the zoospore attachment percentage depending on the particle size (Fig. 2c). We suggest this result was because the area of the minimum gap when the particles were closely arranged on the substrate can be divided into: (1) the area being larger than the full length of a zoospore (ca. 5 µm); and (2) the area being smaller than the full length of a zoospore. When particles of 48.2 µm and more were deposited, the diameter of the inscribed circle that entered the space surrounded by three particles was about 3.7 µm and more, which was almost the same as the full length of the zoospore (diameter ca. 5 µm). That is, when particles of 48.2 µm were densely embedded in a single layer, there was still a substrate to which attachment was possible. However, the gaps between particles of 1.1–15.0 µm were smaller than the full length of the zoospores, so the zoospores attached above the particles. We believe that the attachment of zoospores was significantly inhibited by small amounts of sub-micro particles. We consider that the void area on the gametophyte—that is, the contact area between the gametophyte and seawater—has an effect on survival. Many gametophytes were able to survive even when the percentage gap was 0% (Fig. 2d). This result suggests that, even when the particle layers overlap, it is possible to absorb nutrient salts and gases from seawater in the three-dimensional space between the layers.

ANCOVA analysis was performed on the total length of the gametophytes on day 12 after particle deposition. The total length of female gametophytes was significantly smaller than that of males. Our results also revealed an interaction between particle size and sex. However, Watanabe et al.^26^ found no interaction between sex and particle size, and sex and amount of sediment particles in all gametophytes. This result meant that sex differences appeared in the negative effects of the total length of gametophytes in sub-micro particles.

Watanabe et al.^26^ proposed formulas (Eqs. (7) and (8)), based on this result, to calculate the negative effects of sediment in the range of 15–599 µm on zoospore attachment and the survival of gametophytes of *E. bicyclis*:

7 $$ {\text{Attachment percentage }}A_{r} = 100 \exp ( - 13.5Q/D) { } $$

8 $$ {\text{Survival percentage }}S_{r} = 100 \exp ( - 2.71Q/D) { } $$

where *A*_*r*_ and *S*_r_ denote attachment percentage (%) and survival percentage (%), respectively; and *Q* and *D* show the amount of sediment (mg/cm^2^) and particle diameter (µm), respectively.

A formula (Eq. 9) to calculate the initial depletion *L*_*r*_ of *E. bicyclis* using particle quantity and diameter is derived from Eqs. (7) and (8):

9 $$ L_{r} = 100 [1 - \exp ( - 16.2 Q/D)] $$

**Figure 4:**
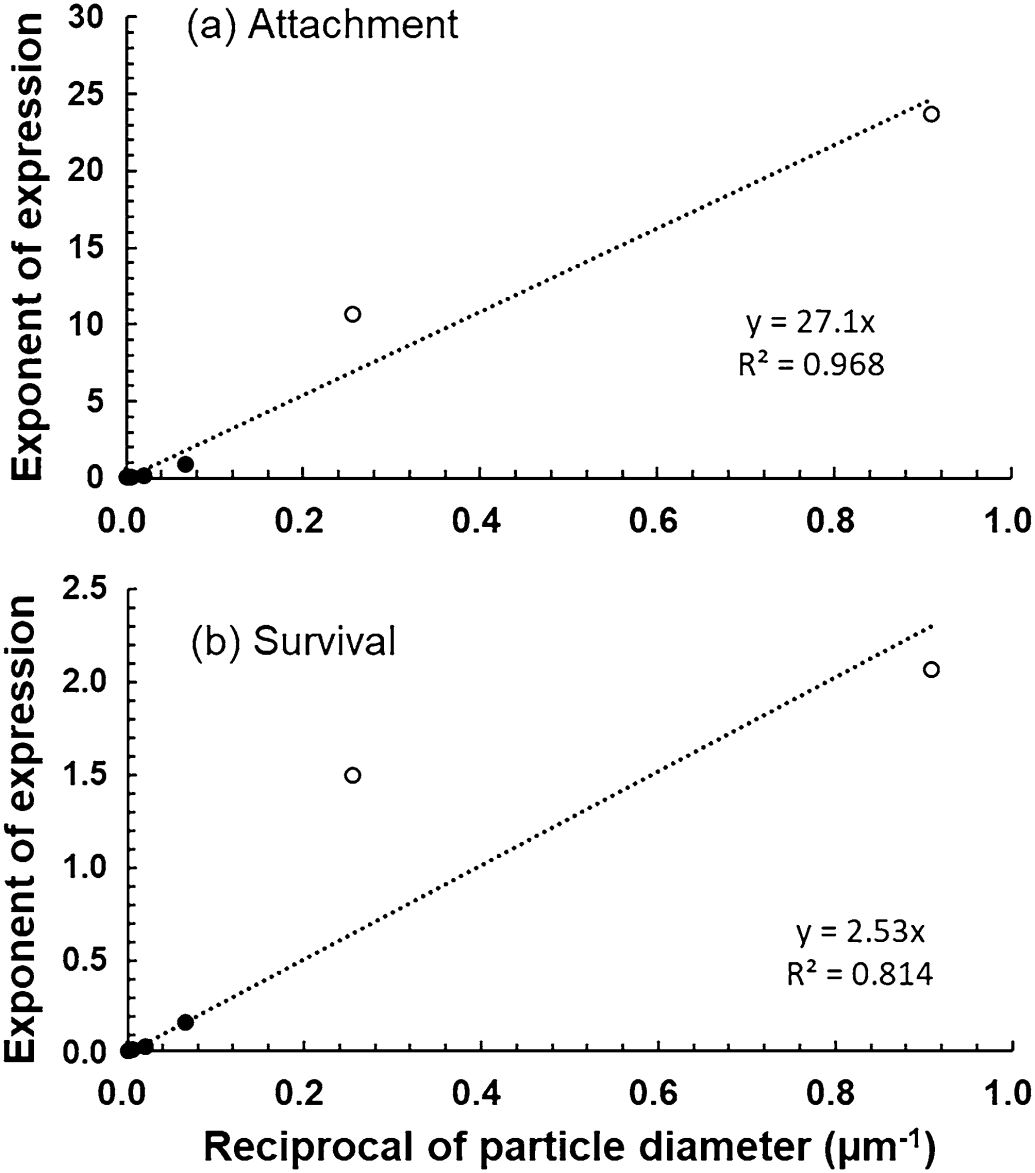
Relationship between reciprocal of particle diameter and exponent of expression. (**a**,**b**) Influence on zoospore attachment and gametophyte survival, respectively. Open circles indicate data on particle sizes 1.1 µm and 3.9 µm in the current study. The data (solid circle) on particle size 15.0–599 µm are calculated from the relational expression of Watanabe et al.^26^.

The results of the current study show that the negative effect of the sub-micro particles was significantly increased. The relationship between the reciprocal of the particle size and the exponent of the relational expression for each particle size is shown in Fig. 4. In this figure, data from the current study were used for particle sizes 1.1 and 3.9 µm, and Watanabe et al.^26^ was used for data of particle size 15–599 µm (Supplementary Table S2 online). The exponent of the relational expression of the attachment percentage gradually increased as the particle size decreased (Fig. 4a). A very good correlation was obtained by exponential approximation in the range of particle size 1.1–599 µm (Eq. (10)):

10 $$ {\text{Attachment percentage }}y = 27.{\text{1x}}, \left( {r^{2} = 0.968} \right) $$

where x is the reciprocal of particle diameter (1/*D*; µm^−1^) and *y* is the exponent of expression for the attachment percentage.

Owing to the influence of the sub-micro size particles, the coefficient of Eq. (10) is much larger than that of Eq. (7) in the range of particles sized 15–599 µm^26^.

The relationship between the reciprocal of particle size and the exponent of expression for the survival percentage (Fig. 4b) is shown in Eq. (11):

11 $$ {\text{Survival percentage }}y = 2.53 x, \left( {r^{2} = 0.814} \right) $$

where x is the reciprocal of particle diameter (1/D; µm^−1^) and y is the exponent of expression for the survival percentage.

In the survival percentage, the coefficient of Eq. (11) differed little from the previous report^26^, owing to the influence of the sub-micro size particles.

By using Eqs. (10) and (11) we were able to obtain the formula for the zoospore attachment percentage and for the gametophyte survival percentage, with the amount of sediment and the particle size as variables (Eqs. (12) and (13)):

12 $$ A_{r} = 100 \exp ( - 27.1 Q/D) $$

13 $$ S_{r} = 100 \exp ( - 2.53 Q/D) $$

where *A*_*r*_ is attachment percentage (%), *S*_*r*_ is survival percentage (%), *Q* is amount of sediment (mg/cm^2^) and *D* is particle diameter (µm).

Using Eqs. (12) and (13), the formula for calculating the initial depletion percentage of *E. bicyclis* owing to sediment particles is Eq. (14):

14 $$ L_{r} = 100 [1 - (A_{r} \times 10^{ - 2} )(S_{r} \times 10^{ - 2} )] $$

The in situ sediments have a wide particle size range. To accurately estimate the influence in that range, it is necessary to divide the particle size into *n* categories and to obtain the product of the influence of each particle, instead of obtaining the influence from the average particle size. The amount of sediment particles in each particle size category can then be used to run Eqs. (15) and (16), and so accurately to calculate the initial depletion percentage:

15 $$ L_{r} = 100 [1 - \exp ( - 29.6 \,{\text{nA}})] $$

16 $$ A = \frac{1}{n} \left( { \frac{{Q_{1} }}{{D_{1} }} + \frac{{Q_{2} }}{{D_{2} }} + \cdots + \frac{{Q_{n} }}{{D_{n} }} } \right) $$

That is, the initial depletion was found to be significantly greater in the presence of sub-micro particles. We investigated the sediment particle quantity and size distribution in Mio, where kelp communities were deforested by particles inflowing from Hidakagawa River in Wakayama Prefecture. We based our examination of the initial depletion in Mio on the results of this survey (Fig. 3) and on Eqs. (15) and (16).

The initial depletion at the zoospore and gametophyte stages in the Mio sea area was estimated to be 94.2%. In contrast, it was 54.7% in the Noshima sea area where kelp communities remain. We considered that the threshold value for determining whether or not a kelp community is formed is between the initial depletion of both values. The initial depletion of sub-micro particles less than 10 µm in Mio was about 75.6%. This indicated that sub-micro particles had a large negative effect in Mio. This study made it possible for us to understand initial depletion on the basis of the particle amount and the size distribution of sediment particles in other sea areas, and to determine the success or failure of the formation of kelp communities.

We estimated the attachment and survival percentage for particles A, B and C (Table S1) by using Eqs. (15) and (16). Comparison of the estimated values with the experiment results revealed that particles A and B had similar results for both values. For particle C, however, the estimated values of the attachment percentage and the survival percentage were extremely small. Particle C comprised many nano-size particles that were not the subject of the current study. We inferred that the effect of nano-size particles differs from those of sub-micro size.

The distribution and behaviour of nano-size particles^37^ on rocky shores where kelp communities are formed are not clear. Investigations into the effects of their chemical toxicity to some organisms is ongoing^31–33^. This study included sub-micro size particles, but did not consider chemical toxicity. We found, however, that the physical effects of sub-micro size particles, irrespective of their chemical toxicity and simply by covering the substrate, were very large. It will, in future, be necessary to study the effects of nano-size particles.

The results of the current study led us to consider that the initial depletion due to sub-micro size sediment particles can be quantified in the sea area by using Eqs. (15) and (16). Our results indicate that the influence of sub-micro size particles cannot be ignored as one of the causes of the decline in marine forests.

When sedimentary particles increase in the canopy-forming kelp forest, the kelp forest is replaced with turf-forming communities. This study quantified the impact of fine particles, including those of sub-micro sizes, and clarified the threshold of seabed sediment particles that generates dynamics leading to a transition from kelp forest to turf communities.

## Methods

### Specimen macrophyte and particles

One of the main marine forest species, *E. bicyclis*, was used for the experiment. *E. bicyclis* sporophytes were collected on the Pacific coast of Chiba, Fukushima, and Ibaraki prefectures, eastern Japan, during the autumn from 2016 to 2018. The blades with zoosporangial sorus spots were cut out, washed thoroughly with sterile filtered seawater and dried in the shade. A zoospore suspension was then prepared by immersing the blades in sterile filtered seawater for 10 min. The concentration range of zoospores was 1 × 10^4^ to 30 × 10^4^ ind./mL, and the suspension was diluted to 1 × 10^4^ ind./mL.

Three types (A, B and C) of SiC particles of different sizes were used as the sub-micro sediment particles. Particles A, B and C were manufactured by Nilaco Co. Ltd (Tokyo, Japan), High Purity Chemical Laboratory Co. Ltd (Saitama, Japan) and Sigma-Aldrich Co. LLC. (Tokyo, Japan), respectively. The sizes of particles A, B and C were measured with the laser diffraction particle size distribution analyser SALD-2300 (Shimadzu Corporation, Kyoto, Japan): the average particle sizes of particles A and B were 1.1 µm and 3.9 µm, respectively (Supplementary Fig. S1 online). Particles A and B had a single peak, and particle C had two peaks. The shapes of particles were mainly spherical or elliptical.

### Zoospore attachment experiment

Various amounts of SiC particles were placed in an Erlenmeyer flask containing 100 mL of filtered seawater, and stirred with ultrasonic waves (USC-12D, Iwaki Glass Co. Ltd, Shizuoka, Japan) for 45–60 min to prepare a liquid in which particles were suspended. A glass slide with a boundary line and a 1-mm grid was placed as the substrate on the bottom of a Nalgene container (outer diameter 61.8 mm, height 125.2 mm), and 100 mL of the particle suspension was poured into it. Different amounts of particles were then deposited on the glass slide with a centrifuge (ca. 600 rpm, about 45 min; Kokusan Co. Ltd, Tokyo, Japan). The amount of sediment was set at 0.03, 0.05, 0.1, 0.2 and 0.3 mg/cm^2^ and no deposition for particles A and B (average particle size 1.1 µm and 3.9 µm). The amount of sediment C (two peaks) was set at 0.01, 0.03, 0.05, 0.1, 0.2, 0.3 mg/cm^2^ and no sediment.

We poured 5 mL of the zoospore suspension containing a concentration of 1 × 10^4^ cells/mL into the container and allowed it to stand for 12 h. The glass slide was removed, its surface was carefully rinsed with filtered seawater and the number of zoospores attached to the glass slide was counted using a microscope (BH-2, Olympus, Tokyo, Japan).

The zoospore attachment percentage was estimated using Eq. (17):

17 $$ A_{r} = \frac{{N_{s} }}{{N_{no} }} \times 100 $$

where *A*_*r*_ is the attachment percentage (%), *N*_*s*_ is the number of zoospores that have attached to the substrate when particles are deposited and *N*_*no*_ is the number of zoospores that have attached to the substrate without particle deposition. The case where there was no particle deposition was taken as a control experiment, and the zoospore attachment percentage was expressed as 100%. The experiments were repeated eight times for particles A and B and 12 times for particle C.

### Gametophyte growth and survival experiment

Six glass slides were placed on the bottom of six Petri dishes (diameter: 15 cm, height: 9 cm) containing 800 mL of sterile filtered seawater (1.5 atm, 121 °C, 35 min). The zoospore suspension was poured so that the number of zoospores in each Petri dish was about 5 × 10^5^. It was allowed to stand for 1 h, and the zoospores attached to the slide. SiC particle suspensions prepared at various concentrations were then added and allowed to stand for 12 h to deposit sediment on the zoospores attached to the substrate. The amount of sediment on the zoospores was 0.1, 0.3, 0.5, 1.0, 5.0 mg/cm^2^ and no sediments in the case of particles A and B; and 0.1, 0.3, 0.5, 1.0, 3.0 mg/cm^2^ and no sediments in the case of particle C.

We placed each slide and attached zoospores with varying amounts of sediments (and a slide without sediment) on the bottom of each Petri dish filled with PESI medium^38^. The Petri dish was placed in an incubator (Taitec Co. Ltd, Saitama, Japan) and cultivation was started. The water temperature in the culture was 20 °C; the light source was a daylight white fluorescent lamp; and the irradiance on the Petri dish was set at approximately 80 µmol photons/m^2^/s. The photoperiod was 12 h (light:dark; 12:12 h). Slides were removed from each Petri dish at 6 days and 12 days after the start of the experiment, and the number of survivors and total body length of the gametophytes were measured by using a microscope (BH-2, Olympus, Tokyo, Japan). The percentage of gametophyte survival was calculated as in Eq. (18):

18 $$ S_{r} = \frac{{N_{s} }}{{N_{no} }} \times 100 $$

where *S*_*r*_, *N*_s_ and *N*_no_ denote the survival percentage (%), the densities of gametophytes after 12 days on slides with sediment particles, and those without sediment particles, respectively. The slides without sediment were taken as control experiments, and the gametophyte survival percentage was expressed as 100%.

The experiment was repeated three times using different batches of spore suspensions for each experimental treatment. We measured the total body length of 60 gametophytes randomly selected on day 6, and 30–40 male and female individuals randomly selected on day 12. Discoloured gametophytes, or those that had fallen off the slide, were considered to be dead.

Correlations between the amount of sediment and the attachment percentage for each particle size were investigated using Spearman’s rank correlations (α = 0.05), as were correlations between the amount of sediment and the survival percentage for each particle size. The approximate expressions by the nonlinear least squares method and the pseudo-determination coefficient were calculated for each particle size, and the negative effect of the particle size and the amount of sediment on attachment percentage and survival percentage was evaluated. Furthermore, to determine the effect of the particle size and the amount of sediment on the total length of gametophytes, analysis of covariance (ANCOVA) was performed. Statistical calculations of attachment percentage, survival percentage and total body length were performed using statistical processing software R (version 3.5.2) (R Core Team, Vienna, Austria) or statistical analysis Statcel Ver. 3 (OMS Publishing Inc., Saitama, Japan).

### Percentage gap on substrate or gametophyte

To investigate the cause of the effect of sedimentary particles on zoospore attachment and gametophyte survival, we estimated the percentage gap between particles. We determined it by subtracting the sum of the maximum cross sectional areas of the particles from the substrate area. The number of particles was estimated from the amount of sediment, particle mean diameter and general density of particles (SiC in the current particle studied; 3.22 g/cm^3^, glass particles in a previous study^26^; 2.5 g/cm^3^). The shape of the sediment particle was assumed to be a sphere. All the particles were allocated an average diameter. The percentage gap was 100% when no particles were deposited on the substrate. When the first layer of the sediment particles was deposited to the full substrate area, it was assumed that the second-layer particles were deposited so as to fill the gaps in the first layer. When the total particle cross sectional area was equal to the area of the substrate bottom, the percentage gap was set to 0%.

### Size distribution and amount of sediment particles on kelp deforestation sea area

We surveyed the properties of seabed sediments off Mio, Wakayama Prefecture, Japan, where large kelp communities are deforested by particles inflowing from the Hidakagawa River (Supplementary Fig. S3 online)^34^. Sta. 1 is the Mio sea area, and Sta. 2 is the Noshima sea area where the kelp communities exist. In October 2018, sediment particles on reefs for kelp^39^ installed on the sea floor in both sea areas (6 m depth) were collected by the airlift method^40^. The sampling area for one sample was 25 cm^2^ in a quadrat. Sampling was performed in three quadrats at each measurement site at the same time. The collected particles were taken back to the laboratory, collected on a filter (pore size: 0.45 µm), desalted with a solution of ammonium carbonate, dried (60 °C, 72 h) and weighed. The particle size distribution was analysed by using a laser diffraction particle size analyser (SALD-2300; Shimazu Inc. Co. Ltd, Tokyo).

## Supplementary information

Supplementary Information.
