# Supplementary material for: Impacts of sub-micrometer sediment particles on early-stage growth and survival of the kelp Ecklonia bicyclis
Source: Sci Rep. 2020 Nov 26;10:20689. doi: 10.1038/s41598-020-75796-x (PMC7693333; doi:10.1038/s41598-020-75796-x)
Supplement: Supplementary file 1 — Supplementary Information. [file 41598_2020_75796_MOESM1_ESM.docx]

**Supplementary online information**

**Impacts of sub-micrometer sediment particles on early-stage growth and survival of the kelp *Ecklonia bicyclis***

Akira Matsumoto^1,2^, Minami Sato^1^ and Hisayuki Arakawa^1 *^

^1^Tokyo University of Marine Science and Technology, 4-5-7 Konan, Minato-ku, Tokyo 108-8477, Japan

^2^ Fukushima Prefectural Research Institute of Fisheries Resources, 1-1-14 Koyo, Soma, Fukushima 976-0005, Japan

*Corresponding author

Address: Tokyo University of Marine Science and Technology, 5-7, Konan-4, Minato, Tokyo 108-8477, Japan

e-mail address: [arakawa@kaiyodai.ac.jp](mailto:arakawa@kaiyodai.ac.jp)

Cover sheet

**Supplementary online information**

**Supplementary Figure S1.** Size distribution of specimen particles.

**Supplementary Figure S2.** Relationship between particle diameter (a) and amount of sediments (b) and gametophyte length for males and females.

**Supplementary Figure S3.** Survey points of quantity and size distribution of seabed sediments.

Sta. 1 is the Mio sea area, and Sta. 2 is the Noshima sea area where the kelp communities exist.

The map was created using the free statistic software R (A Language and Environment for Statistical Computing, R Core Team, R Foundation for Statistical Computing, Vienna, Austria, 2016, https://www.R-project.org) and its additional function “mapdata package” (version 2.2-6).

**Supplementary Table S1.** Comparison of experiment and estimation results.

**Supplementary Table S2.** Exponential expression of relationships between amount of particles and attachment percentage or survival percentage by each particle size. Data of particle size 15–599 µm are those used by Watanabe et al.^26)^.

**Figure S1.** Size distribution of specimen particles.

Lines A, B and C indicate particle size distributions of 1.1 µm, 3.9 µm in mean diameter, and two peaks in mean diameter, respectively.


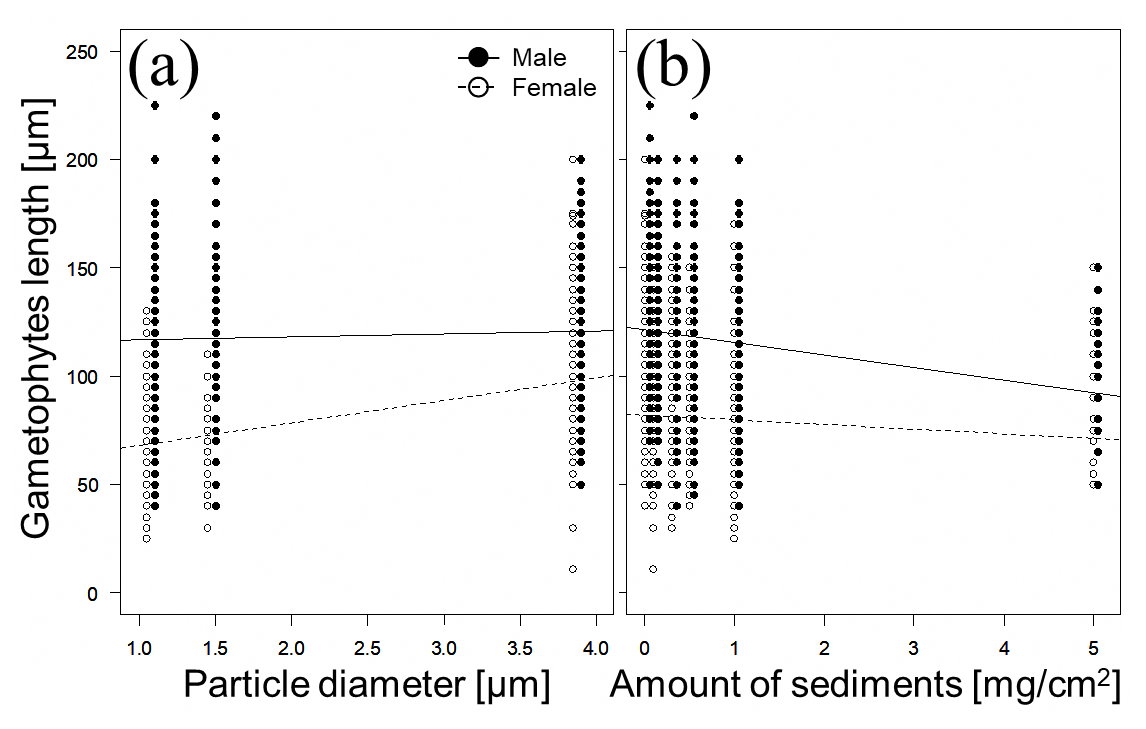


**Figure S2**. Relationship between particle diameter (a) and amount of sediments (b) and gametophytes length for male and female.

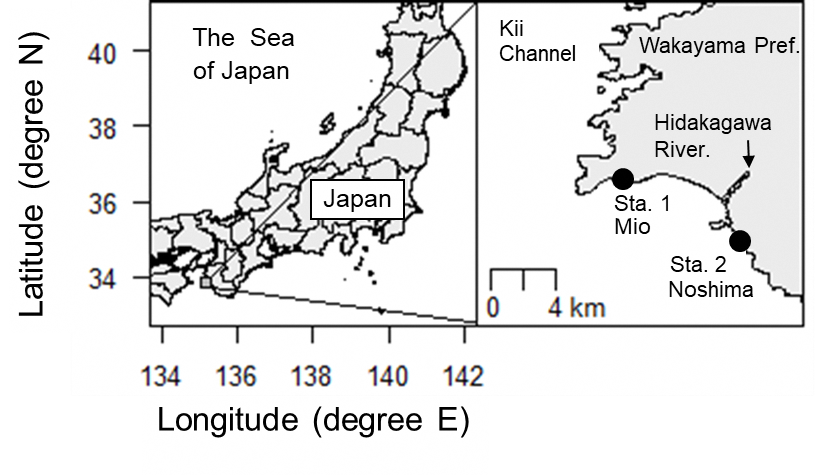


**Figure S3.** Survey points of quantity and size distribution of seabed sediments.

Sta. 1 is the Mio sea area, and Sta. 2 is the Noshima sea area where the kelp communities exist.

The map was created using the free statistic software R (A Language and Environment for Statistical Computing, R Core Team, R Foundation for Statistical Computing, Vienna, Austria, 2016, https://www.R-project.org) and its additional function “mapdata package” (version 2.2-6).

**Table S1.** Comparison of experiment and estimation results.

**Table S2.** Exponential expression of relationships between amount of particles and attachment percentage or survival percentage by each particle size. Data of particle size 15–599 µm are those used by Watanabe et al.^[26]^.
